# Dihydrotanshinone-Induced NOX5 Activation Inhibits Breast Cancer Stem Cell through the ROS/Stat3 Signaling Pathway

**DOI:** 10.1155/2019/9296439

**Published:** 2019-03-24

**Authors:** Su-Lim Kim, Hack Sun Choi, Ji-Hyang Kim, Dong Kee Jeong, Ki-Seok Kim, Dong-Sun Lee

**Affiliations:** ^1^Department of Biotechnology, College of Applied Life Science, Jeju National University, Jeju, Republic of Korea; ^2^Subtropical/Tropical Organism Gene Bank, Jeju National University, Jeju 63243, Republic of Korea; ^3^Aroma Biotechnology Center, Jeju National University, Jeju 63243, Republic of Korea; ^4^Department of Animal Biotechnology, College of Applied Life Science, Jeju National University, Jeju, Republic of Korea; ^5^Department of Internal Medicine, Jeju National University Hospital, Jeju National University, Jeju, Republic of Korea

## Abstract

Cancer stem cells (CSCs) are known to mediate metastasis and recurrence and are therefore a promising therapeutic target. In this study, we found that dihydrotanshinone (DHTS) inhibits CSC formation. DHTS inhibited mammosphere formation in a dose-dependent manner and showed significant tumor growth inhibition in a xenograft model. This compound reduced the CD44^high^/CD24^low^- and aldehyde dehydrogenase- (ALDH-) expressing cell population and the self-renewal-related genes *Nanog*, *SOX2*, *OCT4*, *C-Myc*, and *CD44.* DHTS induced NOX5 activation by increasing calcium, and NOX5 activation induced reactive oxygen species (ROS) production. ROS production reduced the nuclear phosphorylation levels of Stat3 and secreted IL-6 levels in the mammospheres. DHTS deregulated the dynamic equilibrium from non-stem cancer cells to CSCs by dephosphorylating Stat3 and decreasing IL-6 secretion and inhibiting CSC formation. These novel findings showed that DHTS-induced ROS deregulated the Stat3/IL-6 pathway and induced CSC death. NOX5 activation by DHTS inhibits CSC formation through ROS/Stat3/IL-6 signaling, and DHTS may be a promising potential therapeutic agent against breast CSCs.

## 1. Introduction

Breast cancer is a common cancer and a leading cause of cancer death among women [1]. Although widespread mammography and adjuvant therapy with polychemotherapy and tamoxifen for early breast cancer have reduced the mortality of breast cancer [2, 3], breast cancer is the most dangerous disease due to recurrence and metastasis. CSCs were first identified in leukemia [4] and were later found at various solid tumors [5]. CSCs are known as cancer stem-like cells. Additionally, various types of cancer were originated from CSCs [6–8]. This subpopulation changes into tumor through self-renewal and differentiation [9, 10]. The Sonic hedgehog (Shh), Stat3, nuclear factor-*κ*B (NF-*κ*B), Wnt/*β*-catenin, TGF-*β*, and Notch signaling pathways are critical self-renewal factors of CSCs [11–14]. CSCs have drug resistance and radioresistance properties resulting in recurrence of cancer [15]. Therefore, targeting of CSCs is important in cancer therapy. CSCs express specific proteins, including Oct4, Sox2, Nanog, and aldehyde dehydrogenase-1 (ALDH) [16]. ALDH refers to a group of enzymes that oxidizes genotoxic aldehyde, and the ALDH activity is used as a CSC marker of leukemia and head and neck, bladder, bone, colon, liver, lung, pancreatic, prostate, thyroid, and cervical cancers [17]. ALDH is also a therapeutic target of CSCs [18]. The subpopulation of breast cancer expressing CD44^high^/CD24^low^ in clinical specimens had a high capacity to form tumors [19]. Signal transducer and activators of transcription 3 (Stat3) are frequently activated in CSCs, and mammosphere formation is related to the JAK1/2-Stat3 pathway. Secreted IL-6 activates the JAK1/2-Stat3 pathway and upregulates Oct4 gene expression. The IL-6/JAK1/2/STAT3 signaling pathway plays an important role in the conversion of non-stem cancer cells (NSCCs) into CSCs [20]. Blocking the Stat3 signaling pathway inhibits the growth of CD44^high^/CD24^low^ stem cell-like breast cancer cells [21]. The NF-*κ*B transcription factor is constitutively activated in tumor cells, including colon, breast, and liver cancers [22], and is regulated by the I*κ*B kinase (IKK) complex. The NF-*κ*B-specific inhibitor pyrrolidine dithiocarbamate (PDTC) inhibits breast cancer stem-like cells [13].

Reactive oxygen species (ROS) production of cancer cells occurs via multiple mechanisms, and ROS are produced in the mitochondria and by NADPH oxidase (NOX). ROS can play dual functions as important mediators and signal molecules or as damaging factors. As breast CSCs produce lower levels of ROS than tumor cells do, breast cancer stem-like cells are radioresistant [23]. Because ROS are a major mediator of ionizing radiation-induced cell death, CSCs showed less DNA damage than NSCCs did [24]. NOX is a ROS-generating enzyme that can be found not only within the plasma membrane [NOX1–5 and dual oxidase (DUOX) 1-2] but also in the endoplasmic reticulum (NOX2, NOX4, and NOX5), mitochondrial membrane (NOX4), and nuclear membrane (NOX4 and NOX5), as well as in the specialized membrane microdomains caveolin and lipid rafts (NOX1) [25]. NOX5 has Ca^2+^-binding EF hands in the N-terminal. Compared with those of the other NOX members, the function and regulation of NOX5 in tumorigenesis and CSCs are largely unknown.

Dihydrotanshinone (DHTS) is a component of the well-known traditional Chinese medicinal plant *Salvia miltiorrhiza* and is used to treat cardiovascular disease, hepatitis, inflammation, and cancer [26, 27]. Previous studies have shown that DHTS has various biological functions, including liver protection, anti-inflammation, osteoclast differentiation, and tumor cell apoptosis [26, 28–31]. Although DHTS is effective in human cancer cell apoptosis, the exact mechanism of cancer cell apoptosis is poorly understood. In this study, we found that DHTS can selectively inhibit breast CSCs through NOX5/ROS/Stat3/IL-6 signaling and may be a promising potential therapeutic agent against breast CSCs.

## 2. Materials and Methods

### 2.1. Materials

Tissue culture plates, including 6- and 24-well ultralow attachment cluster plates, were obtained from Corning (Tewksbury, MA, USA). DHTS I, crytotanshinone, tanshinone I, and tanshinone II A were purchased from Sigma-Aldrich Co. (St. Louis, MO, USA). Cell growth was assayed using a CellTiter 96® AQueous One Solution kit (Promega, Madison, WI, USA). The ALDEFLUOR™ Kit was obtained from STEMCELL Technologies Inc. (Vancouver, BC, Canada). Chemicals such as M*β*CD were obtained from Sigma-Aldrich Co. (St. Louis, MO, USA).

### 2.2. Cell Culture and Mammosphere Formation

Two cancer cell lines, MCF-7 and MDA-MB-231, were purchased from the American Type Culture Collection (ATCC; Manassas, VA, USA). We followed the method of [32]. Two cancer cell lines were grown in Dulbecco's modified essential medium (DMEM; HyClone, Logan, UT, USA) with 10% fetal bovine serum (FBS; HyClone), 100 U/mL penicillin, and 100 *μ*g/mL streptomycin (HyClone). Two breast cancer cell lines were maintained at 37°C in a humidified incubator with 5% CO_2_. Cells were plated at a density of 1 × 10^6^ cells in T75 culture flasks. For mammosphere formation, single cell-suspended cancer cell lines were seeded at a density of 4 × 10^4^ (MCF-7) and 1 × 10^4^ cells (MDA-MB-231)/well in ultralow-attachment 6-well plates containing 2.5 mL of complete MammoCult™ Medium (Stemcell Technologies). Two breast cancer cell lines were incubated in a 5% CO_2_ incubator at 37°C for 7 days.

### 2.3. Automated Counting of Mammospheres

We followed the method of [32]. At 7 days of culture, a color scale image of the mammospheres was acquired by placing the culture plate on a scanner (PowerLook 1100, UMAX, Seattle, WA, USA). Images at resolutions of 300 or 600 dpi were analyzed using the software program NICE downloaded from https://www.nist.gov/services-resources/software/nists-integrated-colony-enumerator-nice [33]. For counting, regions of interest were created by choosing the desired number of rows and columns (e.g., 2 × 3 for a 6-well plate), and regions of interest were determined by moving and resizing the individual regions after selecting the elliptical setting and the selected images were automatically counted. The mammosphere formation was calculated by the mammosphere formation efficiency (MFE, %), which corresponds to the number of mammospheres per well/the number of total cells per well × 100.

### 2.4. Cell Proliferation

A CellTiter 96® Aqueous One Solution assay kit was used to measure the proliferation rate of MCF-7 and MDA-MB-231 cells. We followed the method of [32] Two breast cancer cells were cultured in a 96-well plate in the presence of DHTS (0.25, 0.5, 1, 2, and 4 *μ*M) for 24 h. We followed the manufacturer's protocol, and the optical density was determined by using a plate reader (Dynex Revelation, Dynex Ltd., Billingshurst, UK). Each series of data were determined in triplicate.

### 2.5. Colony Formation

MDA-MB-231 cells were seeded at a low density in a 6-well plate and treated with 1 *μ*M DHTS. After 1 day, the media were replaced with fresh media and cultured for 10 days. The grown colonies were counted. We followed the method of [34].

### 2.6. Cell Migration

MDA-MB-231 cells were seeded in a 6-well plate and grown at 90% confluency. The scratch was made through the cell layer using a sterile white micropipette tip. After the samples were washed with new media, breast cancer cells were treated with DHTS. We followed the method of [34]. Wounded areas were photographed with a light microscope at 10x after 16 h.

### 2.7. Flow Cytometric Analysis of CD44 and CD24 Expression

Expression of CD44 and CD24 was determined by FACS analysis in MDA-MB-231 cells. We followed the method of [34]. After the cells were harvested and dissociated using 1x Trypsin/EDTA, one million cells were suspended and labeled with FITC-conjugated anti-human CD44 and PE-conjugated anti-human CD24 antibodies (BD Pharmingen, San Diego, CA, USA) and incubated at 4°C for 20 min. Then, the cells were washed three times with 1x PBS and analyzed by flow cytometry (Accuri C6, BD, San Jose, CA, USA).

### 2.8. Measurement of ROS Activity Using DCFDA (2′,7′-Dichlorofluorescein Diacetate) and the CellROX Green Dye Detection Method

Cancer cells were cultured in 96-well cell imaging plates and treated with DHTS (1 *μ*M) or DMSO for 24 h. ROS were detected using DCFDA. DHTS-treated cancer cells were cultured, and the media were aspirated. The cancer cells were washed with 1x PBS and incubated with PBS containing 10 *μ*M DCFDA dye and 5 *μ*M CellROX Green for 30 min at room temperature. DCFDA and CellROX Green dye solution were removed and washed with 1x PBS. Finally, 0.1 mL PBS was added to 96-well plates, and the samples were observed under a phase-contrast fluorescence microscope (Lionheart FX live cell imager, Biotek, Winooski, VT, USA).

### 2.9. Calcium Assay Using the Fluo-4 NW Dye

A Fluo-4 NW calcium assay kit was used to measure the calcium levels of two breast cancer cell lines. The two breast cancer cell lines were grown in a 96-well plate in the presence of DHTS (0, 2, and 4 *μ*M) for 24 h. We followed the manufacturer's protocol (Molecular Probes, Wisconsin, USA), and 100 *μ*L of the dye loading solution was added to a 96-well plate containing breast cancer cells. The plate was incubated at 37°C for 30 min, and the relative fluorescence was recorded using a flow cytometer (Accuri C6, BD, San Diego, CA, USA) and a phase-contrast fluorescence microscope (Lionheart FX live cell imager, Biotek).

### 2.10. Aldehyde Dehydrogenase (ALDH) Activity

ALDH activity was measured using a commercial ALDEFLUOR kit (Stemcell Technologies, Vancouver, BC, Canada). We followed the method of [34]. The ALDH inhibitor diethylaminobenzaldehyde (DEAB) was used as a negative control. The breast cancer cells were treated with 1 *μ*M DHTS for 1 day, and ALDH-positive cells were analyzed with the ALDEFLUOR assay. ALDH-positive and ALDH-negative cells were assayed using flow cytometry (Accuri C6, BD, San Diego, CA, USA).

### 2.11. RT-PCR and Real-Time RT-PCR

Total RNA was isolated using TaKaRa MiniBEST (TaKaRa, Tokyo, Japan) according to the supplier's protocol. We followed the method of [32]. For RT-PCR, 1 *μ*g of total RNA was used for RT-PCR using the One-Step RT-PCR reagent (Qiagen, Germantown, MD, USA). The PCR cycle conditions consisted of 95°C for 0.5 min, 60°C for 0.5 min, and 72°C for 0.5 min followed by a 10 min extension at 72°C. Primers specific to NOX1–NOX5, DUOX1, and DUOX2 mRNAs were as follows: NOX1 primers (P197757_F and P197757; Bioneer, Daejeon, South Korea), NOX2 primers (P301247-F and P301247-R; Bioneer, Daejeon, South Korea), NOX3 primers (P225943_F and P225943_R; Bioneer, Daejeon, South Korea), NOX4 primers (P176436_F and P176436_R; Bioneer, Daejeon, South Korea), NOX5 primers (P210846_F and P210846_R; Bioneer, Daejeon, South Korea), DUOX1 primers (P218762_F and P218762_R; Bioneer, Daejeon, South Korea), and DUOX2 primers (P294520_F and P197757; Bioneer, Daejeon, South Korea). Primers for human *β*-actin, 5′-TGTTACCAACTGGGACGACA-3′ and 5′-GGGGTGTTGAAGGTCTCAAA-3′, were used as an internal control. The levels of transcripts also were determined by a One Step PrimeScript RT-PCR kit using SYBR Green according to the manufacturer's instructions (TaKaRa, Tokyo, Japan). One-step RT-PCR reaction was carried out in 100 ng of total RNA, 10 *μ*L of 2x One Step RT-PCR Buffer, 1 *μ*L PrimeScript 1 step Enzyme Mix, and 10 *μ*M of PCR forward primer and reverse primer of CD 44 (forward: 5′-AGAAGGTGTGGGCAGAAGAA-3′, reverse: 5′-AAATGCACCATTTCCTGAGA-3′), NANOG (forward: 5′-ATGCCTCACACGGAGACTGT-3′, reverse: 5′-AAGTGGGTTGTTTGCCTTTG-3′), OCT4 (forward: 5′-AGCAAAACCCGGAGGAGT-3′, reverse: 5′-CCACATCGGCCTGTGTATATC-3′), SOX2 (forward: 5′-TTGCTGCCTCTTTAAGACTAGGA-3′, reverse: 5′-CTGGGGCTCAAACTTCTCTC-3′), c-Myc (forward: 5′-AATGAAAAGGCCCCCAAGGTAGTTATCC-3′, reverse: 5′-AGCAAAACCCGGAGGAGT-3′), and *β*-actin (forward: 5′-TGTTACCAACTGGGACGACA-3′, reverse primer: 5′-GGGGTGTTGAAGGTCTCAAA-3′) for a final volume of 25 *μ*L per reaction. The relative mRNA expression levels of the target genes were calculated using the comparative CT values. At least four independent PCR procedures were performed to allow for statistical analysis. The PCR products were normalized to that of the *β*-actin gene as an internal control.

### 2.12. Western Blot Analysis

We followed the method of [34]. Proteins isolated from mammospheres treated with DHTS were separated on 10% SDS-PAGE and transferred to a polyvinylidene difluoride membrane (Millipore, Bedford, MA, USA). PVDF membranes were blocked in 3% bovine serum albumin (BSA) in PBS-Tween 20 (0.1%, *v*/*v*) at room temperature for 1 hour. The PVDF membranes were incubated in blocking solution with primary antibodies at 4°C overnight. The primary antibodies used were Stat3, p65, lamin B (Santa Cruz Biotechnology), pStat3 (Cell Signaling, Beverly, MA, USA), and NOX2 (AB Frontier, Seoul, Korea). An antibody against *β*-actin (Santa Cruz Biotechnology) was used as a control. After PVDF membranes were washed with PBS-Tween 20 (0.1%, *v*/*v*), the membranes were incubated with HRP-conjugated secondary antibodies and enhanced using the chemiluminescence detection kit (Santa Cruz Biotechnology, Santa Cruz, CA, USA).

### 2.13. Electrophoretic Mobility Shift Assays (EMSA)

We followed the method of [32]. EMSA was analyzed using a chemiluminescent EMSA kit according to the manufacturer's protocol (Thermo Scientific, IL, USA). The biotin upper and lower strands of the Stat3 probe (5′-CTTCATTTCCCGGAAATCCCTA-Biotin-3′ and 5′-TAGGGATTTCCGGGAAATGAAG-Biotin-3′) were synthesized. Nuclear extracts were isolated from two breast cancer cells as described previously [35]. The biotin-labeled probes were incubated with DHTA-treated nuclear proteins in a final volume of 20 *μ*L EMSA buffer containing poly[dI-dC] for 30 min. The mixtures were electrophoresed on a 6% native PAGE gel in 0.5x TBE at 4°C and visualized using a chemiluminescent nucleic acid detection kit (Thermo Scientific, Waltham, MA USA).

### 2.14. Small Interfering RNA (siRNA)

To determine the effect of NOX2 and NOX5 on mammosphere formation, we treated MDA-MB-231-derived mammospheres with human NOX2 siRNA (NM_000397.3; Bioneer, Daejeon, South Korea) and NOX5 siRNA (NM_001184779.1; Bioneer, Daejeon, South Korea) (or scrambled siRNA control; Bioneer, Daejeon, South Korea). For transfection of the siRNA, the cancer cells were seeded into 6-well plates for 24 h, and transfection was performed using Lipofectamine 2000 (Thermo Scientific) according to the manufacturer's protocol. The levels of NOX2 and NOX5 protein were determined by western blot analyses using anti-NOX2 and anti-NOX5 antibodies.

### 2.15. Chemotherapy of the Breast Cancer Cell-Bearing Immunodeficient BALB/C Nude Female Nude Mice

Female BALB/C nude mice (4–5 weeks old) were purchased from Orient Bio (Seongnam, Korea) and kept in animal facilities for 1–2 weeks. A total of 14 breast cancer cell-bearing BALB/C female nude mice were divided into two groups. The 7 mice in the negative control group received no chemotherapy. However, the volumes of control mouse tumors were measured every three days and then calculated using the formula (width^2^ × length)/2 (Figure 2). Seven nude mice received DHTS using mammary fat pad injection with an optimized dosage of 10–50 mg/kg. All animal experiments and procedures were conducted with a protocol approved by the Institutional Animal Care and Use Committee of Jeju National University. At the end of the experiment, mice were sacrificed, and tumor specimens were taken, photographed, and weighed.

### 2.16. Statistical Analysis

All data are presented as mean ± standard deviation (SD). Data were analyzed using Student's *t*-test. A *P* value lower than 0.05 was considered statistically significant (GraphPad Prism 5 Software, San Diego, CA, USA).

## 3. Results

### 3.1. Effect of Tanshinones on Mammosphere Formation in Breast Cancer Cells

**Figure 1:**
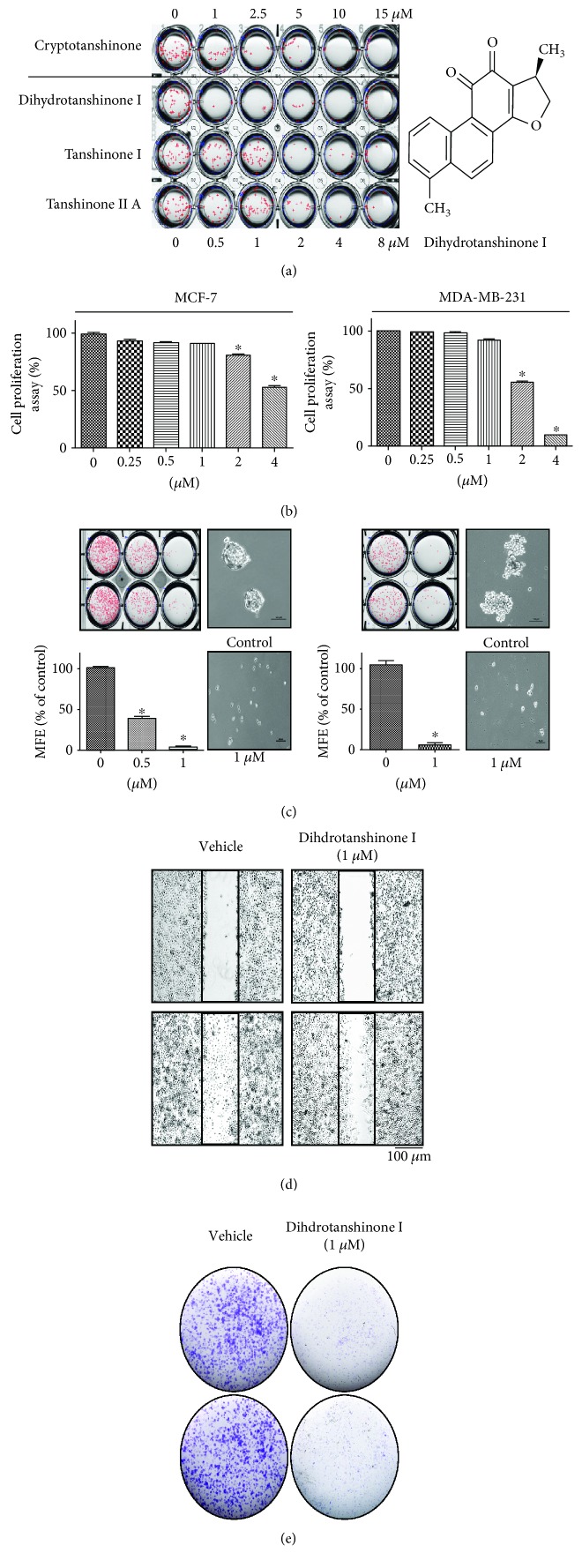
Effect of DHTS on mammosphere formation and multiple cancer hallmarks in breast cancer cell lines. Effect of tanshinones on mammosphere formation. MCF-7 and MDA-231-MB cells were cultured in mammosphere-forming conditions for 7 days. Primary mammospheres were incubated with tanshinones (0, 1, 2.5, 5, 10, and 15 *μ*M) or DMSO. Chemical structure of DHTS (a). Effect of DHTS on the viability of MCF-7 and MDA-MB-231 cells. The two breast cancer cells were treated with DHTS for 24 h. The antiproliferative effect of DHTS was measured by MTS assays (b). Effect of DHTS on mammosphere formation. The mammospheres were incubated with DHTS (0.5 and 1 *μ*M) or DMSO. MCF-7 and MDA-MB-231 cells were treated with DHTS for 7 days in CSC culture media. Images were obtained by microscopy and are representative mammospheres (scale bar = 100 *μ*m) (c). Effect of DHTS on the migratory potential of human breast cancer cells. The wound healing of MDA-MB-231 cells with or without DHTS photographed at 0 and 18 h (scale bar = 100 *μ*m) (d). Effect of DHTS on colony formation of human breast cancer cells. One thousand dissociated MDA-231-MB cells were seeded in 6-well plates and treated with the indicated concentrations of DHTS for 7 days. Representative images of colonies were recorded (e). The data shown represent the mean ± SD of three independent experiments. ^∗^*P* < 0.05 vs. DMSO-treated control.

To evaluate whether tanshinones can suppress the formation of the mammosphere, we added different concentrations of tanshinones to the MCF-7- and MDA-MB-231-derived mammospheres. As shown in Figure 1(a), DHTS produced the most potent inhibitory effect on mammosphere formation. DHTS inhibited the formation of primary mammospheres derived from MCF-7 and MDA-MB-231 cancer cells. Not only were the numbers of mammospheres decreased by 50% to 95% but also the size of the mammospheres was decreased (Figure 1(c)). We examined the proliferative effect of DHTS on two breast cancer cells by MTS assays. There was inhibition of cell proliferation with ≥2 *μ*M (MDA-MB-231 and MCF-7 cells) after 24 h of stimulation (Figure 1(b)). DHTS inhibited the migration and colony formation of MDA-MB-231 cells (Figures 1(d) and 1(e)).

### 3.2. DHTS Inhibits Tumor Growth in a Xenograft Model

**Figure 2:**
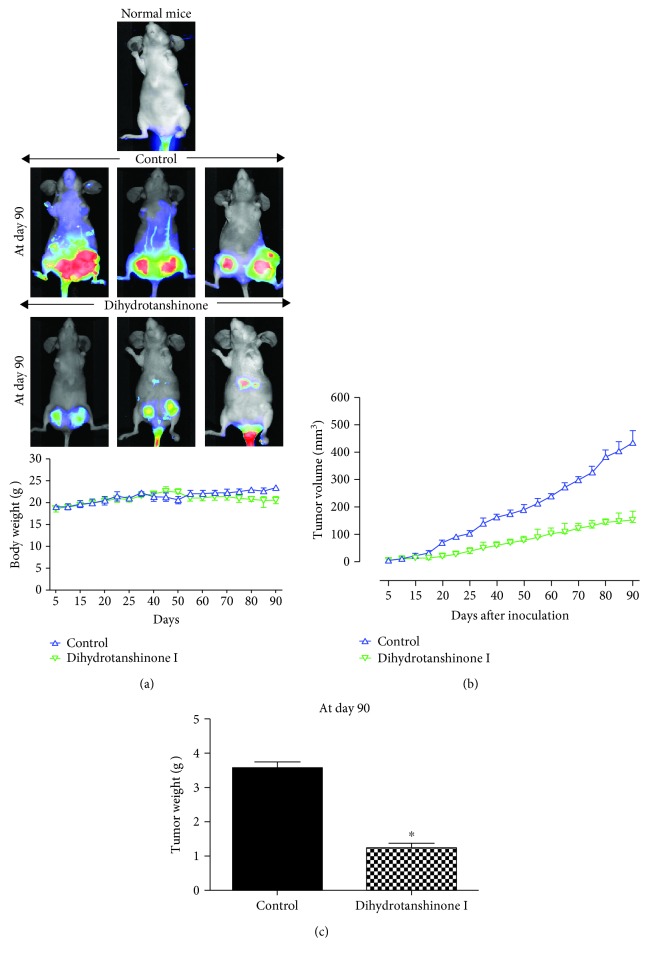
The effect of DHTS on tumor growth in a xenograft model. Three million cells were injected into the mammary fat pad of each immunodeficient NOD-SCID female nude mouse. Analysis of the effect of tumor growth on DHTS and MCF-7 cell-bearing immunodeficient nude mice. The dose of drug used was 10 mg/kg once a week. After 13 weeks, images were captured with an Odyssey® Imager (LICOR, Pearl Image System, USA). The weights of mice were comparable with the control and DHTS-treated groups (a). The high grade of the tumors was detected using the IRDye 800CW optical probe (2DG) in the 800 nm channel, represented in pseudo-color. Tumor volume was measured twice/week using a caliper and calculated as (width^2^ × length)/2. Tumor growth curves were monitored during the experimental period (b). The effect of DHTS on tumor weights. Tumor weights were measured after therapy. ^∗^*P* < 0.05 compared to the control (c). Representative images were captured at the end of 13 weeks of therapy, and the results are shown for vehicle-treated control and DHTS-treated mice.

As DHTS showed antiproliferative effects on breast cancer cells in vitro, we examined whether DHTS inhibited tumorigenicity in a xenograft tumor model. The tumor volume in the DHTS-treated group was smaller than that in the control group (Figures 2(a) and 2(b)). Additionally, tumor weights in the DHTS-treated group were lower than those in the control group (Figure 2(c)). Mice in the DHTS-treated group and control group showed similar body weights (Figure 2(a)). These results demonstrated that DHTS effectively inhibited tumorigenicity in a xenograft model.

### 3.3. Effect of DHTS on Proportion of CD44^high^/CD24^low^- and ALDH-Expressing Breast Cancer Cell Line

**Figure 3:**
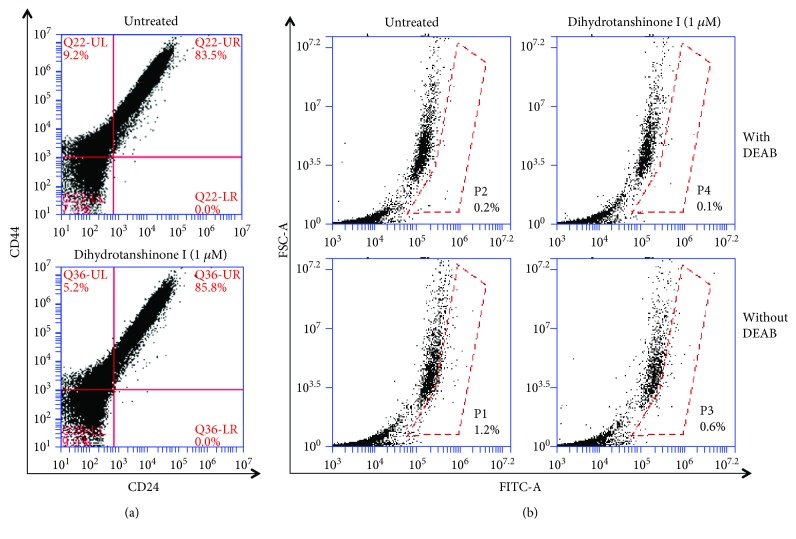
Effect of DTHA on the proportion of CD44^high^/CD24^low^- and ALDH-positive cell in breast cancer cell lines. The CD44^high^/CD24^low^ cell population was analyzed by flow cytometric analysis of MDA-MB-231 cells with DTHA (1 *μ*M) for 2 days. For FACS analysis, 10,000 cells were acquired. Gating was based on a control antibody (Red Cross) (a). The effect of DHTS on the ALDH-positive cell population. MDA-MB-231 cells were treated with DTHA (1 *μ*M) for 2 days and subjected to an ALDEFLUOR assay. The upper panel shows ALDH-positive cells with the ALDH inhibitor, DEAB as a negative control, and the lower panel represents ALDH-positive cells without DEAB. The ALDH-positive population was gated in a box (red dot line box) (b).

MDA-MB-231 cells were treated with DHTS for 1 day, and the CD44^high^/CD24^low^-expressing population of cancer cells was investigated. DHTS decreased the CD44^high^/CD24^low^-expressing population of MDA-MB-231 cancer cells (Figure 3(a)). MDA-MB-231 cells were subjected to an ALDEFLUOR assay to investigate the effect of DHTS on the proportion of ALDH-expressing cancer cells. DHTS decreased the proportion of ALDH-expressing cancer cells from 1.2% to 0.6% (Figure 3(b)). These results showed that DHTS effectively reduced expression of CSC markers.

### 3.4. DHTS Induces ROS Generation, and NAC Reverses DHTS-Induced Mammosphere Inhibition

**Figure 4:**
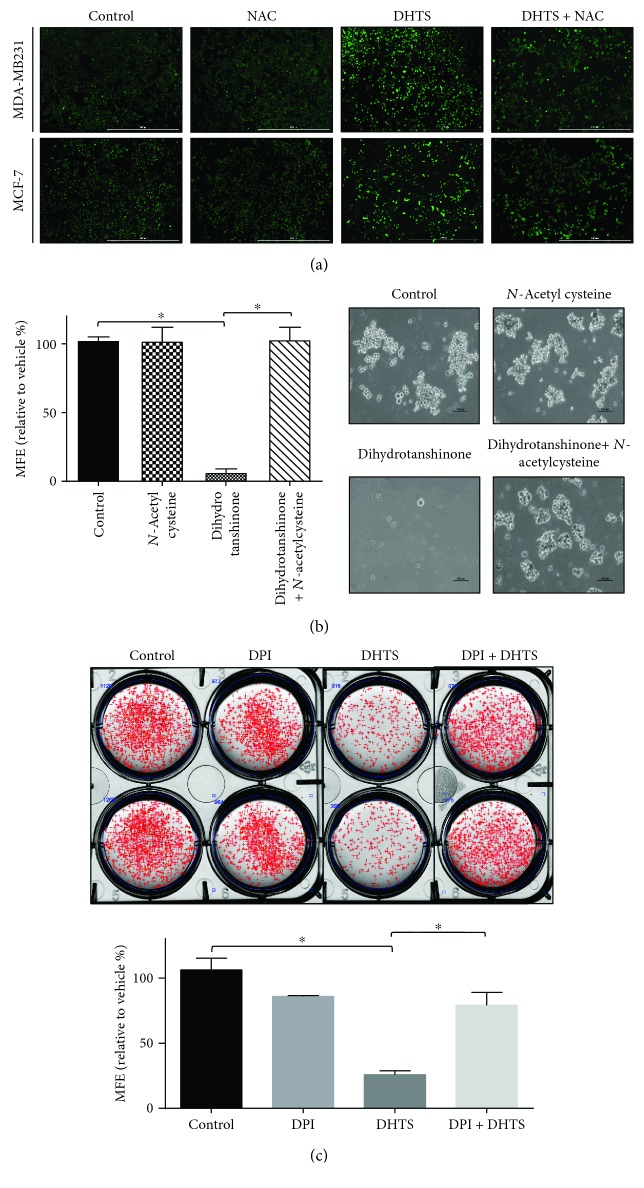
Effect of DHTS-induced ROS on mammosphere formation. The effect of DHTS on ROS generation was determined using DCF-DA assays and fluorescence microscopy. Effect of DHTS on ROS production in MDA-MB-231 cells by DCF-DA staining. Images were obtained by microscopy at 4x magnification and are representative photos (scale bar = 1000 *μ*m) (a). Mammospheres were pretreated with/without NAC (10 mM) 1 h prior to treatment with 1 *μ*M DHTS. After treatment for 7 days, mammosphere formation was determined. Images were obtained by using microscopy at 10x magnification and are representative mammospheres (scale bar = 100 *μ*m) (b). Effect of the NOX enzyme inhibitor DPI (5 *μ*M) on DHTS-induced mammosphere formation inhibition. Mammosphere formation was determined. The dissociated MDA-231-MB cells were seeded in 6-well plates on CSC media and treated with the indicated concentrations of DHTS and DPI for 7 days (c). Representative images of colonies were recorded. The data shown represent mean ± SD of three independent experiments. ^∗^*P* < 0.05 vs. the control.

Generally, increased ROS have been shown to kill CSCs, and low levels of ROS are associated with the stemness of stem cells and CSCs [36]. We examined the levels of ROS in breast cancer cells under DHTS. As shown in Figure 4(a), DHTS induced ROS generation in a concentration-dependent manner in MDA-MB-231 cells. N-Acetylcysteine (NAC) reversed DHTS-induced ROS production. To determine whether DHTS-induced ROS inhibit mammosphere formation, we performed a mammosphere formation assay with NAC. NAC reversed DHTS-induced mammosphere inhibition (Figure 4(b)). ROS production plays an important role in DHTS-induced mammosphere death.

### 3.5. DHTS-Induced Mammosphere Formation Inhibition Is Dependent on NADH Oxidase

To test NOX-dependent ROS production, we determined the effect of the NOX inhibitor diphenyleneiodonium (DPI) on mammosphere formation. DPI pretreatment attenuated DHTS-induced mammosphere inhibition (Figure 4(c)). These results showed that NOX plays a key role in DHTS-mammosphere death and that DHTS is involved in the NOX-dependent pathway.

### 3.6. NOX5, but Not NOX2, Is Involved in DHTS-Induced ROS Production and DHTS-Induced Mammosphere Inhibition

**Figure 5:**
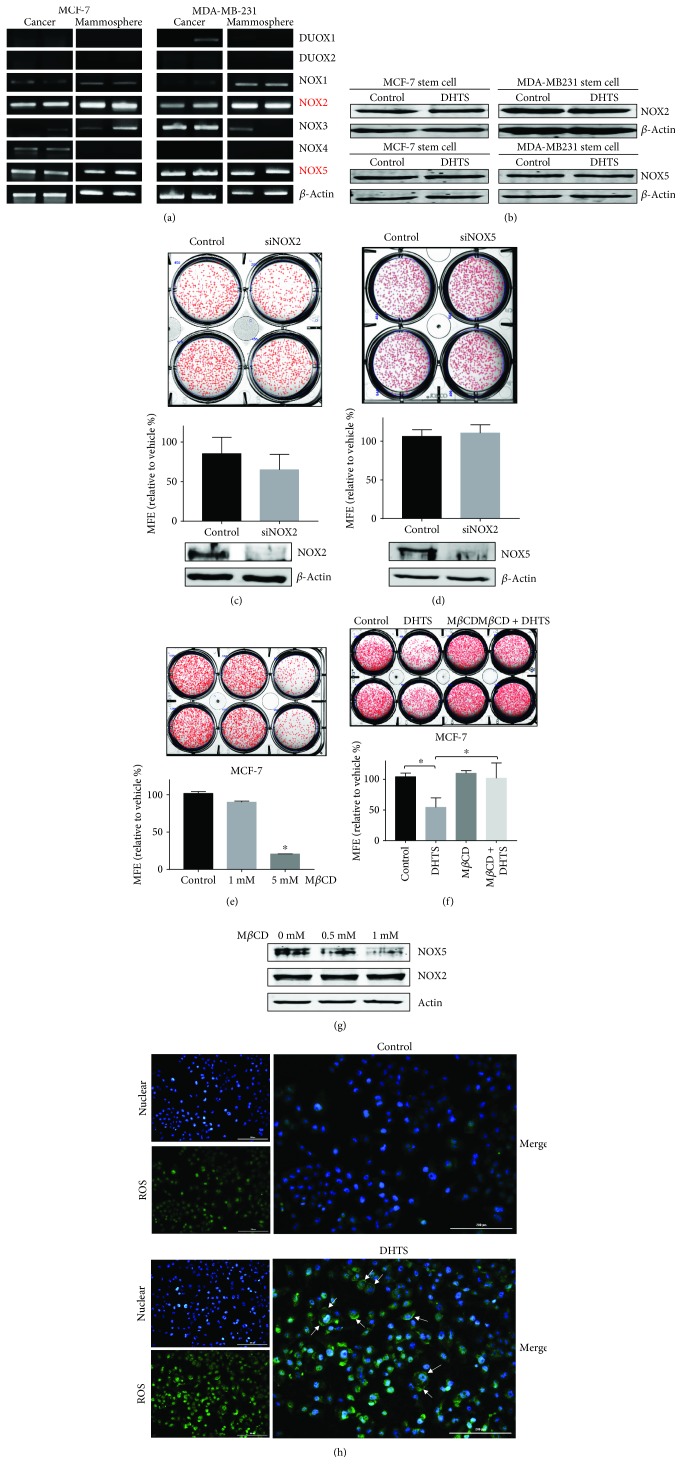
The expression levels of NOX genes and enzymes in breast cancer and CSCs and the effect of the cholesterol scavenger M*β*CD on DHTS-induced mammosphere formation inhibition. The expression levels of NOX gene transcripts in MCF-7 and MDA-MB-231 cells were evaluated using specific primers: NOX isoforms, NOX1–5 and DUOX1–2 primers (a). Mammospheres from MCF-7 and MDA-MB-231 cells were treated with 1 *μ*M DHTS, and the expression levels of NOX2 and NOX5 were evaluated by immunoblot analysis (b). Effect of NOX2 and NOX5 protein on mammosphere formation using siRNA knockdown of NOX2 and NOX5. The mammospheres derived from siRNA-treated cells were cultured for 7 days. Representative images of colonies were recorded. The data shown represent mean ± SD of three independent experiments. ^∗^*P* < 0.05 vs. the DMSO-treated control. NOX2 and NOX5 protein levels were measured by Western blot analyses. All immunoblots are representative of more than 3 independent experiments (c and d). Effect of M*β*CD on mammosphere formation. Mammospheres from MDA-MB-231 cells were treated with 1 mM and 5 mM M*β*CD. Mammosphere formation efficiency was assayed using the control as an M*β*CD-untreated mammosphere (e). Effect of the cholesterol scavenger M*β*CD on DHTS-induced mammosphere formation inhibition. The dissociated MDA-231-MB cells were seeded in 6-well plates on CSC media and treated with the indicated concentrations of DHTS and DMSO for 7 days. Representative images of colonies were recorded. The data shown represent mean ± SD of three independent experiments. ^∗^*P* < 0.05 vs. the DMSO-treated control (f). Effect of M*β*CD on NOX protein depletion in the mammospheres. The mammospheres were treated with 0.5 and 1 mM of M*β*CD for 1 day. Depletion of NOX2 and NOX5 protein was measured by Western blot analyses. All immunoblots are representative of more than 3 independent experiments. (h) Localization of ROS in the cancer cells with/without DHTA (1 *μ*M). ROS were labeled in MDA-MB-231 treated with DHTS or DMSO for 1 day using fluorogenic probe CellROX Green Reagent (5 *μ*mol/L, final concentration). The images were obtained under a phase-contrast fluorescence microscope (Lionheart FX live cell imager, Biotek, Winooski, VT, USA) at 4x magnification and are representative mammospheres (scale bar = 200 *μ*m). Nucleus is blue, and ROS is green. Arrows in lower panels indicated ROS at the plasma membrane (g).

We showed that DHTS regulates mammosphere formation through activation of ROS-generating NOX and then investigated which NOX regulates DHTS-induced mammosphere inhibition. We analyzed the mRNA expression of the NOX isoforms NOX1–5 and DUOX1–2 in MCF-7 and MDA-MB-231 cells and their mammospheres. Our data showed the expression of distinct isoforms of NOX2 and NOX5 in MCF-7 and MDA-MB-231 cells and their mammospheres (Figure 5(a)), and a previous paper showed similar data [37]. The transcripts and proteins of NOX2 and NOX5 were distinctly expressed in breast cancer cells and mammospheres. The major NOX proteins are NOX2 and NOX5, and their protein levels under DHTS were not changed (Figure 5(b)). To test the effect of NOX2 and NOX5 on mammosphere formation, we performed siRNA-inducing silencing of NOX2 and NOX5 expression, which did not reduce the mammosphere formation in MDA-MB-231 cancer cells (Figures 5(c) and 5(d)). These results showed that NOX activation plays a key role in DHTS-induced mammosphere death. To assess DHTS-dependent mammosphere formation inhibition by NOX2 and NOX5, we used methyl-*β*-cyclodextrin (M*β*CD) for a functional study of NOX2 and NOX5. As a cholesterol-sequestering agent, M*β*CD can selectively and rapidly remove cholesterol and cause disruption of lipid rafts [38]; this molecule has been reported to possess free radical scavenging activity and to protect against oxidative stress caused by the addition of hydrogen peroxide [39]. The NOX enzyme activity of breast cancer was decreased by M*β*CD [40]. To select a proper concentration of M*β*CD for the mammosphere formation assays, we added M*β*CD to the mammospheres. We found that 1 mM of M*β*CD did not affect mammosphere formation, but 5 mM of M*β*CD dramatically inhibited mammosphere formation (Figure 5(e)). We tested the effect of DHTS on mammosphere formation using 1 mM M*β*CD. The inhibitory effect of DHTS on mammosphere formation was ameliorated by M*β*CD. These data showed that DHTS-induced ROS production was ameliorated by M*β*CD. To explain the relationship between NOX and ROS production, we tested the release of NOX proteins from the mammospheres. Our data showed that M*β*CD decreases the NOX5 protein level, but not the NOX2 level, in a concentration-dependent manner, and DHTS-dependent mammosphere formation inhibition is related to ROS production by NOX5 enzyme activation (Figures 5(f) and 5(g)).

### 3.7. Localization of ROS under DHTS in MDA-MB-231 Cells

To test the localization of ROS after DHTS treatment in breast cancer cells, we added DHTS to the cells and stained the cancer cells with the fluorogenic probe CellROX Green Reagent to visualize ROS. We examined the levels of ROS in breast cancer cells under DHTS. As shown in Figure 5(h), DHTS induced ROS generation in MDA-MB-231 cells. To determine where DHTS-induced ROS are located, we merged the ROS Green Reagent with the Hoechst nuclear dye. Our data showed that ROS are localized at the cell membrane (Figure 5(h)).

### 3.8. DHTS Triggers Mammosphere Death by ROS-Induced Calcium Release

**Figure 6:**
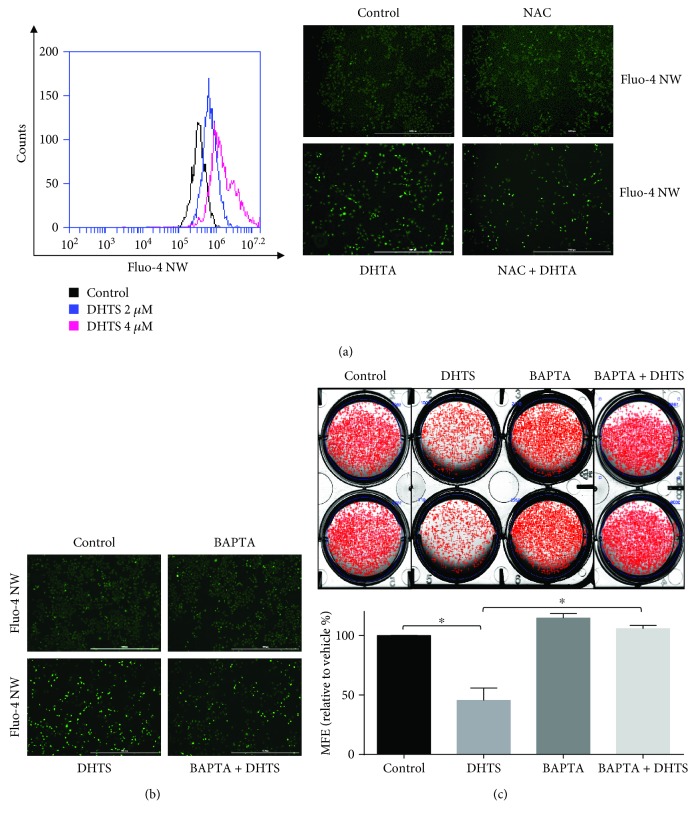
DHTS triggers CSC death by increasing calcium release. DHTS induced calcium release, and DHTS-induced calcium release was ameliorated by NAC. Breast cancer cells were treated with 2 and 4 *μ*M DHTS, and calcium release was assayed using a calcium assay kit containing Fluo-4 AW reagent. After DHTS treatment, cancer cells were stained with Fluo-4 AW, and calcium release was detected by fluorescence microscopy (4x) (scale bar = 1000 *μ*m) (a). DHTS induced calcium release, and DHTS-induced calcium release was ameliorated by the calcium chelator BAPTA. Breast cancer cells were treated with DHTS with/without BAPTA, cancer cells were stained with Fluo-4 AW, and calcium release was detected by fluorescence microscopy (4x) (scale bar = 1000 *μ*m) (b). DHTS-induced calcium release inhibited CSC formation, and its inhibition was ameliorated by a calcium chelator. The dissociated MDA-231-MB cells were seeded in 6-well plates on CSC media and treated with the indicated concentrations of DHTS and BAPTA for 7 days. Representative images of colonies were recorded. The data shown represent mean ± SD of three independent experiments. ^∗^*P* < 0.05 vs. the DMSO-treated control (c).

Activation of the NOX5 enzyme requires calcium [41]. We assessed calcium release under DHTS treatment using the Fluo-4 NW calcium assay method. DHTS induced calcium release from breast cancer cells in a concentration-dependent manner (Figure 6(a)). DHTS-induced calcium release was ameliorated by NAC (Figure 6(a)). These data showed that DHTS induced calcium release and then ROS production. We examined whether DHTS inhibits mammosphere formation using the calcium chelator BAPTA-AM. The number of DHTS-treated mammospheres was decreased, and DHTS and BAPTA-AM treatment ameliorated the inhibition of mammosphere formation (Figures 6(b) and 6(c)). These data showed that DHTS triggers mammosphere death by ROS-induced calcium release.

### 3.9. Effect of DHTS on the Stat3 Pathway Activation and Secreted IL-6 in Mammospheres

**Figure 7:**
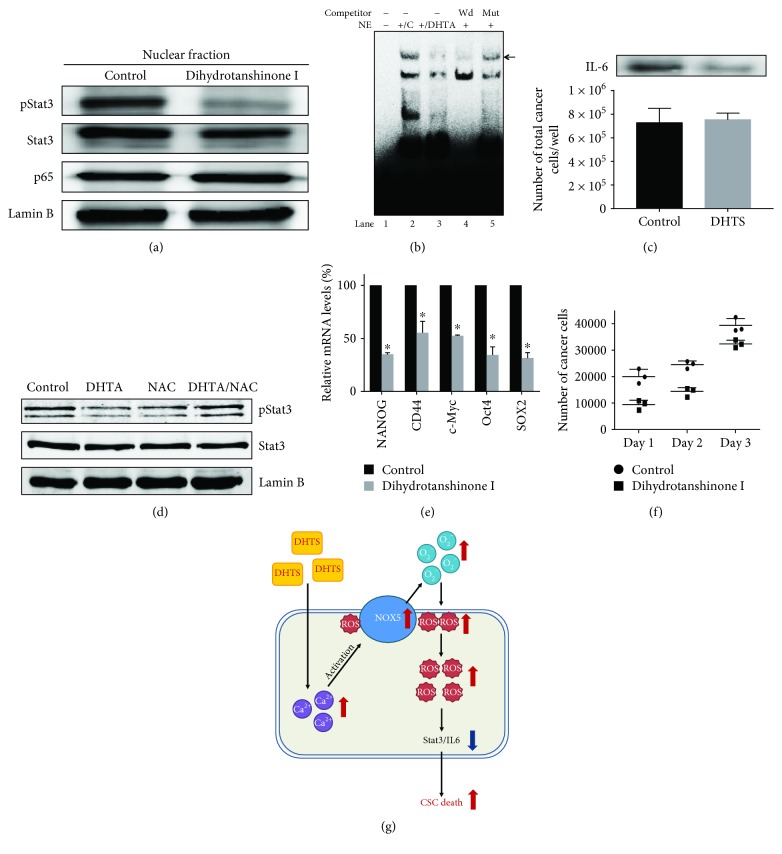
Effects of DHTS on the Stat3 pathway activation in the mammospheres and CSC loads in breast cancer. The activation of Stat3 and NF-*κ*B was determined in the mammospheres with antibodies to pStat3, Stat3, p65, and lamin b. DHTS decreased the nuclear pStat3 level in the mammospheres (a). EMSA assay using the mammosphere lysates treated with DHTS. Nuclear lysates were incubated with a Stat3 probe and separated by 6% PAGE. Lane 1: probe only; lane 2: nuclear extracts with probe; lane 3: DHTS-treated nuclear extracts with probe; lane 4: self-competition; lane 5: nuclear extracts incubated with mutated Stat3 probe (b). Immunoblot of extracellular fluids from mammosphere cultured solution with an anti-IL-6 and the number of cancer cell/well as internal control (c). The effects of DHTS and NAC on pStat3 phosphorylation. DHTS-induced dephosphorylation of pStat3 was ameliorated by NAC (d). Transcriptional levels of the CSC markers Nanog, Sox2, Oct4, c-Myc, and CD44 were determined in DHTS-treated mammospheres using gene-specific primers and real-time RT-PCR. *β*-Actin acts as an internal control (e). Effect of DHTS on mammosphere growth. DHTS prevented mammosphere growth. The DHTS-treated mammospheres for 2 days were dissociated into single cells and plated in 6 cm dishes with equal numbers of cells. Twenty-four hours after plating, the cells were counted. At 2 and 3 days after, the cells were counted in triplicate and plotted as the mean value. The data shown represent mean ± SD of three independent experiments. ^∗^*P* < 0.05 vs. the control (f). The proposed model for CSC death by DHTS. DHTS induced calcium release and activated NOX5, and then NOX5 produced ROS. ROS induced dephosphorylation of Stat3 and reduced secreted IL-6. The secreted IL-6 can convert NSCCs to CSCs and regulate dynamic equilibrium from NSCCs to CSCs. DHTS deregulates equilibrium from NSCCs to CSCs through dephosphorylation of Stat3 and deregulation of IL-6 and kills CSCs (g).

The ROS-mediated activation of the STAT3 signaling pathway was involved in cellular senescence [42]. To determine the biological function of DHTS, we examined the Stat3 and NF-*κ*B pathways in mammosphere-derived MDA-MB-231 cells under DHTS treatment. DHTS can reduce the nuclear pStat3 protein level compared to DMSO treatment. But DHTS could not reduce the nuclear p65 protein level (Figure 7(a)). We did EMSA to analyze Stat3/DNA binding using a Stat-binding oligonucleotide that binds Stat3 protein [43]. As shown in Figure 7(b), DHTS reduced the ability of Stat3 protein to bind the Stat3 probe (Figure 7(b), lane 3). The specificity of the pStat3/Stat probe oligonucleotide was assayed using an excess unlabeled oligonucleotide self-competitor (Figure 7(b), lane 4) and a mutated-Stat oligonucleotide competitor (Figure 7(b), lane 5). These data suggest that DHTS inhibits the Stat3 signaling pathway. IL-6 cytokine plays an important role in the formation of mammosphere [44]. To determine the level of secreted IL-6, we did Western blot analysis in mammosphere-cultured solution by using an anti-IL-6. After DHTS treatment, Western blot data indicated that DHTS reduced the level of secreted IL-6. An internal control was used to count numbers of cancer cell/well with/without DHTS (Figure 7(c)). To determine whether DHTS induces dephosphorylation of Stat3, we examined pStat3 levels with NAC. NAC could reverse DHTS-induced dephosphorylation of Stat3 (Figure 7(d)). These results showed that ROS/Stat3 signaling plays a key role in DHTS-induced mammosphere death.

### 3.10. Effect of DHTS on Transcriptional Levels of Self-Renewal Gene in CSCs and Proliferation of Mammospheres

To test whether DHTS regulates the transcriptional levels of self-renewal genes, we examined the transcriptional levels of self-renewal genes using by real-time RT-PCR. DHTS reduced the expression of self-renewal genes of Nanog, Sox2, Oct4, c-Myc, and CD44 in CSCs (Figure 7(e)). To assay whether DHTS regulates mammosphere growth, we treated DHTS on mammospheres, and cancer cell numbers were counted. DHTS killed cancer cells in the mammospheres, and a decreased cancer cell number was observed in DHTS-treated mammospheres. DHTS led to a decrease in mammosphere proliferation (Figure 7(f)).

## 4. Discussion

Breast cancer is one of the major causes of morbidity and mortality in women. Although mortality of breast cancer has decreased due to widespread mammography screening and adjuvant therapy containing tamoxifen and chemotherapy, this cancer is a fatal disease in females [45]. The CSCs are involved in self-renewal and differentiation, similar to normal stem cells, and contribute to drug resistance of cancer patients [46]. The original CSC research was mainly based on leukemia stem cells. Solid tumor stem cells derived from breast cancer were isolated for the first time in 2003 [19]. Many CSCs were isolated from cancer patients and cancer cell lines. As CSCs from cancer patients are limited, CSCs of cancer cell lines were useful in cancer research.

Our results show evidence for DHTS as an antitumor and anti-CSC agent of breast cancer: (1) DHTS inhibited the proliferation of breast cancer cells and the size and formation of the mammospheres (Figure 1); (2) DHTS inhibited cell migration and colony formation of cancer cells (Figure 1); (3) DHTS suppressed tumor growth of mice in vivo (Figure 2); (4) DHTS reduced the CD44^high^/CD24^low^ and ALDH-positive populations in cancer cells (Figure 3); (5) DHTS inhibited mammosphere formation through ROS production, and DHTS-induced mammosphere inhibition was ameliorated by NAC (Figure 4); (6) A NOX inhibitor, DPI, ameliorated DHTS-induced mammosphere inhibition. The major NOX proteins of breast CSCs are NOX2 and NOX5, and DHTS induced mammosphere formation inhibition, which is dependent on NOX5 activation by calcium release (Figures 5 and 6). (7) DHTS inhibited the Stat3 signal pathway of the mammospheres (Figure 7). DHTS reduced the secretory IL-6 level, which is an important cytokine of CSCs (Figure 7) [47], and induced a decrease in mammosphere growth (Figure 7). DHTS can be an anticancer agent by targeting breast cancer and CSCs.

Recent studies have shown that DHTS inhibits breast adenocarcinoma through G1 arrest and apoptosis and induces ROS-dependent apoptosis in colon cancer cells [26, 48]. CSCs in human and mouse breast tumors have lower ROS levels than the corresponding nontumorigenic cells [49], and this low level of ROS seems to be associated with high expression of ROS-scavenging molecule [50]. CSCs have a high antioxidant capacity to maintain cellular ROS and a strongly increased antioxidant property for CSC survival and drug resistance.

The NOX family is a group of transmembrane proteins able to transport electrons from NADPH and to reduce oxygen to the ROS superoxide anion (O_2_^·−^) and hydrogen peroxide (H_2_O_2_) [51]. In mammals, NOX1 to NOX5 and DUOX1 and DUOX2 comprise the seven NOX proteins [25]. Our data showed that NOX proteins of breast CSCs are primarily NOX2 and NOX5. DTHS-induced CSC inhibition occurs via the NOX5 enzyme. NOX5 activation is dependent on EF hands (helix-loop-helix motifs) that bind calcium ions [52]. For the first time, we showed that NOX5 activation is essential for breast CSC death (Figure 6). ROS generated by NOX enzymes induced activation of Stat3 and autophagy of cancer cells [53]. We assessed the Stat3 signal after DHTS-induced ROS production. DHTS inhibited the Stat3 signal of CSCs and IL-6 production (Figure 7). The Stat3 signaling pathway is a critical factor in the function of normal stem cells and plays an important role in breast CSCs. The nuclear translocation of phosphorylated Stat3 by DHTS was tested in breast mammospheres. We showed that DHTS reduced the nuclear translocation of phosphor-Stat3 (Figure 7(a)). Breast CSCs are resistant to different anticancer agents and responsible for breast cancer relapse. The constitutively activated phosphor-Stat3 is responsible for 30–60% of primary breast cancer. Our data indicated that DHTS reduced the protein levels of nuclear pStat3 and Stat3 DNA-binding activity. DHTS inhibited formation of mammosphere via the blocking of the Stat3 signaling pathway (Figure 7).

Tumors composed of multicellular types are heterogeneous: cancer cells, CSCs, cancer-associated fibroblasts, macrophages, and tumor-infiltrating lymphocytes. Solid tumors have CSCs and NSCCs, and equilibrium between CSCs and NSCCs is controlled by secreted IL-6 [47]. The IL-6 antibody blocked the CSC formation [44]. The cytokine IL-6 and IL-8 regulated self-renewal of CSCs [54]. Blockade of secretory IL-6 using anti-IL-6 can be a useful tool for targeting CSCs. DHTS inhibited the secretion of IL-6 and can be a candidate for inhibition of IL-6 secretion in solid tumor (Figure 7). IL-6 is important in the formation of mammospheres. Because the presence of secretory IL-6 is correlated with a poor prognosis in cancer patients, the regulation of intratumoral IL-6 is important for cancer treatment [55].

DHTS is a naturally occurring compound extracted from *S. miltiorrhiza*, a Chinese medicinal plant, and has been reported to have cytotoxicity to a variety of tumor cells. DHTS has been shown to induce apoptosis via induction of endoplasmic reticulum stress [56]. DHTS can target breast CSCs by blocking inflammatory pathways containing NOX5/ROS/Stat3 signaling and IL-6. Our data showed that DHTS may be used as a new breast cancer chemopreventive agent.

## Data Availability

The data used to support the findings of this study are available from the corresponding author upon request.

## Additional Points

*Highlights*. Dihydrotanshinone (DHTS) induced NOX5 activation by increasing calcium, and NOX5 activation induced reactive oxygen species (ROS) production. ROS production reduced the nuclear phosphorylation levels of Stat3 and secreted IL-6 levels in the mammospheres. DHTS deregulated the dynamic equilibrium from non-stem cancer cells to CSCs by dephosphorylating Stat3 and decreasing IL-6 secretion and inhibiting CSC formation. These novel findings showed that DHTS-induced ROS deregulated the Stat3/IL-6 pathway and induced CSC death. NOX5 activation by DHTS inhibits CSC formation through ROS/Stat3/IL-6 signaling, and DHTS may be a promising potential therapeutic agent against breast CSCs.
